# Mitigation strategies to control a carbapenem-resistant *Acinetobacter baumannii* outbreak in a dedicated COVID-19 unit

**DOI:** 10.1017/ash.2022.125

**Published:** 2022-05-16

**Authors:** Candace Campbell, Calvin White, Carolee Estelle

## Abstract

**Background:** Carbapenem-resistant *Acinetobacter baumannii* (CRAb) is considered a public health threat, and this pathogen is typically associated with hospital infections. At 3 months after opening the hospital’s dedicated COVID-19 unit, 2 patients were identified with CRAb. Infection prevention staff collaborated with staff in the COVID-19 unit, hospital leadership, and health department partners to develop mitigation strategies and to prevent additional transmission. **Methods:** Admissions to the COVID-19 unit were stopped. Biweekly surveillance cultures were collected to identify any patients potentially colonized with CRAb. An infection control risk assessment was conducted to determine breaches in infection prevention practices. The risk assessment included environmental rounding of the area, epidemiological investigation, environmental testing, pulsed-field gel electrophoresis (PFGE) testing, and observing infection prevention practices. **Results:** The risk assessment identified multiple gaps in infection control practices, for example, gaps in hand and environmental hygiene practices. The extended use of personal protective equipment (PPE), staff shortages, fatigue, and staff taking on multiple roles and tasks outside their general job duties were other gaps identified by the team. Between June and September 2020, 43 additional CRAb cases were identified in the facility, with 4 (9.8%) cases outside the COVID-19 unit. Moreover, 29 cases (64%) were considered clinical infections and 16 (36%) were identified from surveillance efforts. Environmental cultures identified 1 positive surface with CRAb. PFGE testing was completed on 44 isolates; 42 isolates had identical PFGE patterns, and 2 isolates were unrelated to the COVID-19 unit; 2 isolates were closely related (with 3-band differences) but were not identified in the COVID-19 unit. **Conclusions:** The inability to definitively identify the source of transmission made it challenging to determine the best approach to eradicating the pathogen. Mitigation for outbreaks should focus on not deviating from core infection control practices.

**Funding:** None

**Disclosures:** None

